# The neurobiology of openness as a personality trait

**DOI:** 10.3389/fneur.2023.1235345

**Published:** 2023-08-14

**Authors:** Maison Abu Raya, Adedoyin O. Ogunyemi, Jake Broder, Veronica Rojas Carstensen, Maryenela Illanes-Manrique, Katherine P. Rankin

**Affiliations:** ^1^Global Brain Health Institute, University of California, San Francisco, San Francisco, CA, United States; ^2^Memory and Aging Center, Department of Neurology, Weill Institute for Neurosciences, University of California, San Francisco School of Medicine, San Francisco, CA, United States; ^3^Department of Community Health and Primary Care, University of Lagos, Lagos, Nigeria; ^4^Neurogenetics Research Center, Instituto Nacional de Ciencias Neurologicas, Lima, Peru

**Keywords:** Big Five model of personality, openness, dogmatism, cognitive flexibiliity, neurobiology

## Abstract

Openness is a multifaceted behavioral disposition that encompasses personal, interpersonal, and cultural dimensions. It has been suggested that the interindividual variability in openness as a personality trait is influenced by various environmental and genetic factors, as well as differences in brain functional and structural connectivity patterns along with their various associated cognitive processes. Alterations in degree of openness have been linked to several aspects of health and disease, being impacted by both physical and mental health, substance use, and neurologic conditions. This review aims to explore the current state of knowledge describing the neurobiological basis of openness and how individual differences in openness can manifest in brain health and disease.

## Introduction

Openness is one of the major personality traits derived from the Big Five model, which is a widely accepted framework for understanding personality that also includes the factors conscientiousness, extraversion, agreeableness, and neuroticism. Openness is characterized by a person’s tendency to seek out new experiences and to be willing to explore ideas, values, emotions, and sensations that differ from their previous experience or established preferences (1). This trait has been extensively studied in the field of personality psychology and has been associated with a variety of positive outcomes, such as increased creativity, curiosity, adaptability, mental flexibility, and acceptance of others (2).

For instance, openness can boost creativity because individuals who score high in openness tend to be more imaginative and original in their thinking and, thus, are more likely to be receptive to new and unconventional ideas, which in turn can inspire them to think outside the box and come up with innovative solutions to problems (3). Research has shown that greater openness is directly associated with enhanced creative achievement (4). Openness is also related to mental flexibility, which refers to the ability to adapt one’s thinking and behavior to better fit with changing situations and contexts. Individuals who are high in openness tend to be more adaptable, allowing them to navigate uncertain and complex situations with greater ease (5). Trait openness is also connected to more effective, innovative, and ethical leadership because studies have shown that open leaders are more likely to be receptive to feedback and new information, have better critical thinking and quicker problem-solving capabilities, make better decisions, and be more empathically responsive to the needs and mistakes of their followers (6, 7).

Openness as a personality trait has been widely studied in cultural and organizational psychology as well (8). While trait openness correlates with individuals’ career advancement into managerial and professional roles (9), studies show that individuals who score high in openness are better able to manage conflicting cultural values and adapt to new cultural contexts, which is a crucial factor underlying success in multicultural organizations and environments. Openness has also been associated with various positive outcomes in professional settings, including better job performance, organizational citizenship behavior (10), and intercultural competence (11).

## Measuring openness

Studies have generally used one of two approaches for measuring openness: standardized self- and other-report questionnaires, or direct neuropsychological measures in which openness is conceptualized to overlap with creativity and thus is measured via the volume and quality of creative output.

Typical questionnaire measures of openness ask individuals to rate themselves on a series of items related to their openness to new experiences, ideas, and ways of thinking (12). One of the most comprehensive standard questionnaires for assessing openness is the NEO personality inventory (NEO-PI) (13), which is a popular personality assessment tool that measures the “Big Five Personality Traits” including trait openness. This tool was developed by McCrae and Costa and was first published in 1978 (1). Over the years, they have published three updated versions of the inventory, with the latest being the NEO PI-3 in 2005 (13–15). It measures openness via 68-item subscale facet scores addressing openness to fantasy, aesthetics, feelings, actions, ideas, and values. The fantasy facet refers to the individual’s level of imagination, creativity, and tendency to indulge in daydreams. The aesthetics facet assesses the individual’s appreciation of art, music, and beauty. The feelings facet refers to the individual’s emotional awareness, sensitivity, and tendency to experience deep and intense emotions. The actions facet refers to the individual’s level of adventurousness, willingness to take risks, and preference for novelty. The ideas facet assesses the individual’s level of intellectual curiosity, open-mindedness, and appreciation for new ideas. Lastly, the values facet refers to the individual’s level of openness to alternative belief systems, such as spiritual or religious beliefs (14).

Another commonly used measure of openness is the HEXACO personality inventory (16), developed by Lee and Ashton in the early 2000s as an alternative to the NEO PI-R. It measures six broad dimensions of personality: honesty-humility, emotionality, extraversion, agreeableness, conscientiousness, and openness to experience. The HEXACO model differs from the NEO PI-R in that it includes the honesty-humility dimension and places a greater emphasis on the ethical and moral aspects of openness. The inventory has been revised to improve the psychometric properties of the measure, resulting in the 100-item HEXACO-PI-R in 2018 (4, 17, 18).

The California psychological inventory (CPI) is a personality inventory developed by Gough and Bradley in the 1950s, currently in its most recent version being the CPI-434, which was released in 2005 (19). The CPI is a self-report assessment tool that measures personality traits on 20 scales, including dominance, sociability, responsibility, self-control, and tolerance. The California psychological inventory (CPI) does not have a specific scale for openness; however, the CPI does assess several dimensions investigators have argued are conceptually related to openness, including intellectual efficiency, creativity, and aesthetic appreciation (20).

Another widely used method for measuring openness is through creativity tests that include mental tasks which require individuals to think in unconventional ways. For example, the torrance tests of creative thinking (TTCT) was created by Torrance in the late 1950s and has been studied longitudinally for more than five decades to further validate the test across all age groups (21). This composite test assesses figural and verbal creativity using various subscales such as fluency, originality, elaboration, abstractness of thought, resistance to premature closure, and flexibility, which are based on the performance of different tests that need divergent thinking and other problem-solving skills (22). These tasks are designed to elicit imaginative and original responses from participants, and many of them involve generating alternative uses of objects, making associations between seemingly unrelated items, or imagining hypothetical scenarios (22). Additional examples of these types of “challenge” tasks include the alternative uses task (AUT) (22), the remote associates test (RAT) (23, 24), and the consequence task (25, 26), all of which require individuals to engage in divergent thinking, making distant associations and generating consequences for unlikely or impossible events.

## The neurobiological basis of openness in neurologically healthy persons

Studies have used a mixed array of neuroimaging techniques in conjunction with these personality inventories with the goals of localizing the neural networks responsible for shaping openness as a personality trait, and developing better insight into the cognitive mechanisms that anchor openness neurobiologically. The majority of studies have sought to establish a correlation between scores on these measures and different brain regions by examining specific patterns of brain structure and function or neurochemical activity in individuals measured to have different levels of openness. Neuroimaging techniques such as structural magnetic resonance imaging (sMRI), resting-state functional MRI (rsfMRI), magnetic resonance spectroscopy (MRS), single photon emission computed tomography (SPECT), and positron emission tomography (PET) have each provided insights into the neurobiological basis of openness via this group comparison approach. A smaller set of studies have attempted to examine openness through task-based neuroimaging methods such as fMRI, in which participants were asked to perform a task in the scanner that the investigators construed as reflecting cognitive or emotional openness, such as the TTCT, AUT, or RAT. This research design might be less directly applicable to understanding openness as a personality trait but may still shed light on the specific neurobiology of cognitive processes known to contribute to openness, such as creativity and mental flexibility (22, 27–30).

Together, these studies have demonstrated distinct patterns of brain structure, connectivity, and activity, as well as neurochemical correlates, in brain regions known to be associated with creativity, abstraction, and cognitive flexibility. Broadly, the neurobiology of openness seems to be supported by three main functions and their corresponding neural networks: (1) reward processing, including the dopaminergic system along with ventromedial frontal and limbic reward networks, (2) the capacity to identify others’ perspectives and distinguish them from one’s own, which is supported by the brain’s default mode network, and (3) higher-order reasoning and decision-making, which is mediated by the executive frontoparietal control network (ECN). Evidence suggests that higher connectivity and functional integration among these three systems predicts trait openness. A summary of findings from neuroimaging studies across different methodologies is shown in Table 1.

**Table 1:** Summary of neuroimaging studies on trait openness.

Study	Sample	Openness questionnaires/other tasks	Imaging modality/network or region analysis methodology	Results summary and interpretation
DeYoung et al. (2010)	116 healthy individuals	NEO-PI-R^2^	sMRI^1^ Whole brain volumes and ROIs^3^ (voxel-level) expansion or contraction compared to the reference image	There were no discernible correlations between openness and local brain volume One cluster in the right parietal cortex was linked to this feature but was too small to cross the cluster-size criteria (2)
Riccelli et al. (2017)	507 healthy participants from the human connectome study	NEO-FFI^4^	sMRI^1^ SBM^5^	Greater area and folding in the prefrontal-parietal regions and a thinner cortex were associated with openness. These results show a relationship between individual variance in the sociocognitive dispositions outlined by the FFM and anatomical variability in prefrontal cortices (30)
Bjørnebekk et al. (2012)	265 healthy individuals	NEO-PI-R^2^ BDI^6^ WASI^7^	sMRI^1^ Multimodal imaging approach: regional analysis of cortical morphometry and white matter DTI^8^	The personality trait most directly connected to brain shape was neuroticism Greater neuroticism was linked to decreased total brain volume, extensive WM microstructure loss, and reduced frontotemporal surface area The inferior frontal gyrus was narrower in people with higher extraversion ratings, and the temporoparietal junction was adversely correlated with conscientiousness There were no conclusive links between agreeableness and openness and brain anatomy (31)
Wenfu Li et al. (2015)	246 college students	NEO-PI-R^2^ RAPM^9^ WCAT^10^	sMRI^1^ VBM^11^	These findings suggest that an individual’s trait creativity may be significantly influenced by the specific personality trait of openness to experience and that creativity and the appropriate pMTG volume are related through openness to experience to some extent (34)
Yasuno et al. (2017)	37 healthy participants	NEO-FFI^4^	sMRI^1^ VBM^11^	Variations in intra-cortical myelination in the anterior cingulate/medial frontal cortex, posterior cingulate cortex, and posterior insula/adjacent putamen are related to individual differences in openness to experience These results support the theory that myelination serves as a biological underpinning for the trait of openness and plays a role in the relationship between creativity and mental illnesses (32)
Marstrand-Joergensen et al. (2021)	295 unique healthy individuals	NEO-PI-R^2^	^12^rsfMRI Resting-state functional connectivity	Openness, including the fantasy component, was inversely correlated with DMN functional connectivity in the resting state (35)
Wang et al. (2022)	376 healthy participants	NEO-PI-R^2^ Creativity tasks: ^13^CAQ, ^14^CBI, ^15^BICB Divergent thinking tasks: ^16^PIT, ^17^AUT, ^18^UST	^12^rsfMRI Specific networks functional connectivity analysis Including the dorsal and ventral attention network, default mode network, limbic network, control network, and two others for somatosensory and visual networks	At the behavioral level, there is a correlation between creative achievement and both experiential openness and diverse thinking. Both openness to new experiences and divergent thinking involves the attentat networks and the default mode network since they both call for focus and the capacity for spontaneous thought (27)
Sun et al. (2019)	29 healthy university students	^2^NEO-PI-R Divergent thinking tasks: ^17^AUT, ^19^OCT (as a control task)	Task-fMRI Activation functional connectivity analysis	Different combinations of network connectivity patterns predict creativity and openness to experience Positive connections between the precuneus and supramarginal gyrus and the middle frontal gyrus/superior frontal gyrus were found Individual difference analysis showed a significant correlation between openness to experience and the intensity of functional connectivity between various important default mode, cognitive control, and salience network areas The network-based mechanisms that underlie creativity and the neurological foundation of individual differences in openness to experience were found to be true (54)
Wei et al. (2014)	269 healthy individuals	Divergent thinking: measured by the torrance tests of creative thinking	Pre- and post-task—resting state fMRI Whole-brain voxel-based activity and ROI-functional connectivity	Study findings suggest that increased RSFC between the default mode network’s mPFC and mTG may be essential for creativity and that cognitive stimulation can increase RSFC between these two brain regions (reflecting creativity training-induced changes in functional connectivity, especially in the lower creativity individuals who had lower scores of torrance tests of creative thinking) (55)
Beaty et al. (2018)	163 healthy adults	Creative ideation task, alternate uses task (AUT) of divergent thinking	Two task-based fMRI samples and one task-free resting-state sample fMRI during creative ideation task Functional connectivity analysis	Greater default mode network, SN, and ECN functional connectivity are associated with higher creativity and divergent thinking (56)

A summary of findings and methodology from neuroimaging studies showing correlational structural and functional connectivity and activity to variability in openness and creativity and divergent thinking as another aspect of trait openness. We conducted a comprehensive literature search using the PubMed database for studies published from 1979 to 2023 in peer-reviewed journals that investigated the neurobiological correlates of openness. We included original articles that reported brain imaging data or neurophysiological measures of brain function in relation to measures of openness. Here we review the most recent relevant neuroimaging studies. ^1^Structural MRI (sMRI), ^2^Revised NEO personality inventory (NEO-PI-R) (57), ^3^Region of interest (ROI), ^4^NEO-five-factors-inventory (NEO-FFI) (13), ^5^Surface-based morphometry (SBM), ^6^Beck depression inventory (BDI) (58), ^7^Wechsler abbreviated scale of intelligence (WASI) (59), ^8^Diffusion tensor imaging (DTI), ^9^Raven’s advanced progressive matrix (RAPM) (60), ^10^The creativity assessment packet (WCAT) (61), ^11^Voxel-based morphometry (VBM), ^12^Resting-state functional magnetic resonance imaging (rsfMRI), ^13^The creative achievement questionnaire (CAQ) (62), ^14^Creative behavior inventory (CBI) (63), ^15^The biographical inventory of creative behaviors (BICB) (64), ^16^The product improvement task (PIT), ^17^The alternate uses task (AUT) (21), ^18^The utopian situations task (UST), ^19^Object characteristics task (OCT).

### Structural neuroimaging studies

The correlation between regional brain volumetrics and openness in healthy individuals has been examined in several studies with varying results. While some structural neuroimaging studies have found no relationship between openness and cortical brain volume (31), others have implicated a diverse set of structures. DeYoung and colleagues found that individuals who score high in openness tend to have a larger prefrontal cortex, which is the part of the brain responsible for higher-order thinking, decision-making, and planning (2). They also found an association with the volume of the inferior parietal lobule, which is linked with working memory, attention control, and general intelligence, suggesting that these cognitive processes might be associated with openness (2).

Openness has also been associated with increased gray matter volume in the anterior cingulate cortex (ACC), which is involved in emotion regulation and conflict monitoring (32).

Another neuroimaging study of healthy older adults from the Baltimore longitudinal study of aging found that higher openness was associated with increased gray matter volume in the frontopolar cortex. These regions are involved in cognitive control and executive function (33, 34), and enable individuals with higher openness to hold alternative actions in working memory in order to evaluate new options and ideas (33). The same study showed negative correlations between openness and volume in the right ventromedial prefrontal (vmPFC) and left fronto-insular cortex, regions that are involved in evaluation of negative outcomes and are linked to inhibitory or cautionary reactions to unpleasant or threatening stimuli. These data suggest that people with higher trait openness might be less vulnerable to such inhibitory reactions, while individuals with higher levels of anxiety are less likely to engage in cognitive or behavioral openness due to perceived risk (33, 35).

### Functional connectivity studies

Two networks, in particular, appear in the majority of functional connectivity studies of creativity. The first, typically called the default mode network (DMN), includes the posterior cingulate cortex (PCC), dorsomedial prefrontal cortex (dmPFC), lateral parietal cortex, and hippocampal memory regions (33, 36). This network is involved in self-referential processing and social perspective-taking and is thought to be involved in a range of cognitive processes, including interpersonal perspective taking, introspection, and autobiographical memory (37). The second network relevant to trait openness is the executive control network (ECN), which comprises the dorsolateral prefrontal cortex (dlPFC) and the lateral posterior parietal cortex (PPC). It is an externally-oriented network involved in attentional selection, active task control, and executive functions, and is responsible for higher-order reasoning and decision-making. The dlPFC has also been implicated in the regulation of affect via reallocation of attention (36, 38).

Task-free functional imaging studies suggest that openness may be associated with increased functional connectivity *between* brain networks involved in cognitive control (i.e., the ECN) and self-referential processing (the DMN) (27). While higher levels of activity within the DMN have been found to predict lower trait openness, higher levels of connectivity between the DMN and ECN appear to allow individuals with higher levels of openness to better process information, generate new ideas, and approach challenges in creative and innovative ways. This DMN-ECN connectivity pattern in individuals with higher levels of trait openness is also correlated with cognitive flexibility, allowing these individuals to switch between different mental sets and think outside the box (38). Furthermore, evidence from these studies suggests that connectivity of the DMN and ECN networks with the brain’s reward regions (including the vmPFC, nucleus accumbens, and head of the caudate) allows individuals with higher trait openness to be better able to integrate diverse sources of emotional and cognitive information, and to have a greater propensity to turn self-reflection and introspection about emotionally salient ideas and experiences into creative action (35).

In otherwise neurologically healthy individuals, certain cognitive and behavioral approaches and personality traits can be maladaptive, even falling on the spectrum of psychopathology. Dogmatism, or fundamentalism, can be understood to represent the opposite of trait openness because it is characterized by rigid adherence to a set of ideas and the intentional exclusion of competing beliefs. Thus, studies of dogmaticism are relevant to the neurobiology of openness, and have important implications for the mechanisms of decision-making, problem-solving, and learning (28). Task-based fMRI studies have shown that individuals with high levels of mental rigidity exhibit lower activation in the vmPFC during tasks that require flexible thinking, such as set-shifting or task-switching, compared to individuals with high levels of mental flexibility (28, 39). Dogmatism has been found to be associated with decreased functional connectivity between the vmPFC and the temporoparietal junction (TPJ) (28), while ideologic openness is associated with increased functional connectivity between the vmPFC and the ACC, regions involved in error monitoring and motivation (28, 40, 41).

### Neurochemistry

One of the key neural systems that has been implicated in trait openness is the dopaminergic mesolimbic pathway, which is involved in reward processing, motivation, and novelty-seeking behavior. Several lines of research have demonstrated that individuals high in openness exhibit greater activation in the ventral striatum, a key component of the mesolimbic reward pathway, during tasks that involve processing novel or unexpected information. Other studies showed that individuals who have a particular variant of the dopamine receptor gene (DRD4) tend to score higher in openness (42). The DRD4 gene has also been linked to sensation-seeking behavior and risk-taking (43, 44).

Similarly, the neurotransmitter serotonin is associated with emotion regulation, and individuals who have the long allele variant of the serotonin transporter gene (5-HTTLPR) tend to score higher in openness (45). The short allele variant of the 5-HTTLPR gene has also been linked to anxiety and depression (46), which are negatively correlated with trait openness. Studies have examined the relationship between openness and the serotonergic system using positron emission tomography (PET) with different serotonergic receptor-binding radioligands, but with varied results. Kalbitzer and colleagues showed that participants’ scores on the NEO-PI-R openness scale, and particularly the two subscales openness to actions and openness to values, were negatively correlated with the [11C] DASB binding radioligand for 5-HTT in limbic areas including the caudate (47). The authors suggested that because there was less serotonin available in these areas, the action of the remaining serotonin was potentiated, similar to the facilitation caused by antidepressant treatment with selective serotonin reuptake inhibitors (SSRIs). However, other PET studies using different serotonin ligands [5-HT2AR (48) and 5-HT4R (49)] to investigate trait openness found no significant neural associations.

Certain drugs, such as psychedelics (i.e., LSD and psilocybin, which are agonists for the 5-HT2AR serotonin receptor), have been shown to increase openness in some individuals (50, 51). fMRI studies have also shown altered DMN activity and connectivity (52) in individuals during psychedelic use. As described earlier, the DMN is involved in self-referential thinking and introspection, leading to better self-awareness and self-regulation, though heightened DMN activity alone corresponds with decreased openness (36). However, the pattern of brain activity induced by psychedelics is characterized by increased variability and decreased stability, which may in turn result in greater connectivity between the DMN and other networks. The authors posit that this leads to a “liberation” of cognitive and affective processes, allowing for increased creativity and divergent thinking as part of the trait openness (52).

Research using magnetic resonance spectroscopy (MRS) has also revealed that individuals with high levels of openness have higher levels of the neurotransmitter glutamate in anterior cingulate cortex (ACC) regions related to error monitoring and motivation, as well as in vmPFC reward areas (53). Glutamate is a key neurotransmitter involved in synaptic plasticity and learning, and these results suggest that increased glutamatergic activity in these reward and motivation areas in more open individuals may lead to a greater capacity for cognitive flexibility and learning (53).

### Trait openness in neurologic disease

While the studies described thus far have included predominantly healthy individuals, additional insights about the neuroanatomical and neurochemical basis of openness can be derived from research models of neurological diseases as well. Lesion studies can shed light on which brain areas are both necessary and sufficient for particular behaviors and thought processes. Studying altered neural activity and connectivity in individuals with brain aging, injury, and disease who also show atypical levels of trait openness can facilitate our understanding of the underlying neural mechanisms.

### Age-related cognitive decline

Studies have investigated how trait openness relates to aging as and age-related cognitive decline. Several investigations have found a negative correlation between openness and age (11, 15, 65); however, others showed that openness can have a protective effect against cognitive decline in middle-aged and older adults (66, 67) and correlates with better cognitive performance, social abilities, and well-being in older age (66, 68). Other research has suggested that openness may represent a behavioral channel to cognitive and social engagement, which are linked to a lower risk of dementia and cognitive decline (13, 69, 70).

### Traumatic brain injury

Studies of individuals with severe structural brain lesions (28, 71), such as penetrating traumatic brain injury (pTBI) (28), show that they often exhibit an extreme lack of openness in the form of mental rigidity, dogmatism, and ideological or religious fundamentalism. Brain-behavior studies of these individuals’ patterns of mental rigidity highlight the role of the vmPFC and its connectivity with other brain regions (28). In a study that included a large sample of patients with pTBI, Zhong and colleagues found that patients with injuries to the vmPFC scored higher than patients with dlPFC lesions on a standardized scale of religious fundamentalism, and that on average both groups showed abnormally high scores compared to neurologically healthy individuals. Analyses adjusting for the size of the lesions in the vmPFC suggested the interaction between vmPFC and dlPFC drove patients to have less cognitive flexibility and openness, again supporting the idea that the connection between reward- and executive-processing areas supports trait openness. This study gives insight into the role of both vmPFC and dlPFC in the revision of religious beliefs, suggesting that loss of cognitive flexibility is linked to an increase in fundamentalist belief adherence and resistance to novel information (28).

### Neurodegenerative disease

The neurobiological mechanisms underlying changes in openness in individuals with neurodegenerative brain disease are complex and are still being elucidated. Similar to what has been observed in neurologically healthy individuals, studies in individuals with neurodegenerative disease have suggested that changes in the levels of dopamine, serotonin, and other neurotransmitters may play a role (54, 72). Additionally, the changes in frontal and temporal brain structure and connectivity often observed in patients with neurodegenerative disorders have been repeatedly linked to dysregulation and alteration of previously stable personality traits (73).

Several studies have suggested that individuals with neurodegenerative disorders such as Alzheimer’s disease (AD) and Parkinson’s disease (PD) may experience a decline in openness, particularly with respect to creativity and decision-making (74). Parkinson’s disease is characterized by the degeneration of dopaminergic neurons in the substantia nigra that can lead to both motor and non-motor symptoms. Individuals with PD are more likely to have reduced openness to experience, the degree of which has been associated with the severity of motor symptoms and cognitive decline (75). In PD, atrophy in the vmPFC and dlPFC have been shown to impact both social cognition and decision-making, leading to a decrease in intellectual curiosity (76).

Notably, there is a growing evidence that some personality traits increase the likelihood of developing Alzheimer’s disease and other dementias (77, 78). Several studies showed that openness, as a premorbid personality trait, was related to better cognitive outcomes in later life, suggesting that openness to experience contributes to cognitive reserve (79, 80). Openness also correlates with lower levels of aging-related hippocampal volume loss (81), and less Alzheimer’s disease-related tau accumulation in the entorhinal cortex in cognitively healthy individuals (82).Tautvydaite and colleagues found that in a mixed group of individuals with and without AD-positive biomarkers, premorbid openness predicted cognitive performance regardless of the individual’s cognitive level, demographics, APOEε4 status, or CSF biomarker levels. They found that openness was the only personality domain from the five-factor model that contributed independently to cognitive performance (83). These findings imply that openness as a lifelong personality trait may play a protective role against age-related neuropathological processes (33, 79, 82).

Another neurodegenerative disorder that has been shown to directly impact trait openness is frontotemporal dementia (FTD). FTD is characterized by the degeneration of the frontal and temporal lobes of the brain, leading to changes in behavior, personality, and language (84). Research has shown that individuals with the behavioral variant FTD syndrome (bvFTD) have significantly lower scores on measures of openness compared to healthy controls (85, 86), and that this decline has been linked to neurodegeneration in regions including the vmPFC and ACC (87).

Mental rigidity and dogmatism are common symptoms of FTD, and can manifest in various ways. For example, many individuals with FTD exhibit perseveration, which is the repetition of the same behavior or thought despite changes in the environment. Studies suggest this behavior may be due to atrophy in the dlPFC and ACC, areas involved in cognitive flexibility and inhibitory control (88). Some patients may present with intense resistance to changes in their routine or environment, such as trying new foods, moving to a new residence, or wearing different clothes day to day. Again, studies have linked this with atrophy in the dlPFC and other brain regions that support cognitive flexibility and adaptive behavior (89). Other patients with FTD, particularly those with the right temporal or semantic variant of bvFTD (90), may hold rigid or inflexible beliefs and refuse to consider alternative viewpoints (91, 92). Evidence suggests this may be due to disruptions in the vmPFC, which is involved in making evaluations of rewards during decision-making (93, 94). Furthermore, disruptions in the white matter tracts connecting the dlPFC and other brain regions such as the insula and anterior cingulate cortex, correlate with cognitive inflexibility and dogmatism in FTD (95).

On the other hand, studies of individuals with FTD have also highlighted the relationship between openness and creativity, which can be unleashed in a subset of these patients. For instance, specific patients with bvFTD have been found to display enhanced creativity and divergent thinking in specific contexts such as artistic production (96–98). A subset of individuals with FTD may exhibit dramatically increased engagement in an artistically creative behavior, such as painting, drawing, or composing music, that was not present before the onset of the disease (85, 99). This enhanced creative production is thought to be related to changes in the brain network connectivity between the DMN and the salience network (SN) (95). This phenomenon has been referred to as “unleashed creativity” and may be related to changes in neural networks involved in the processing of semantic and emotional information (96).

An alternate theory to explain this phenomenon is that the loss of inhibitory control that occurs in FTD may release previously suppressed creative tendencies (100). Certain patterns of dysfunction in the vmPFC and dlPFC may lead to a shift in cognitive processing that favors creative thinking over other, more rigorous cognitive processes (99). Persons with FTD who show this unleashed creativity show greater atrophy in the left hemisphere of the brain, particularly in the ventral and dorsolateral frontal and temporal regions associated with social cognition, executive functioning, and semantic processing. This atrophy appears to release inhibitions in the creative process, allowing for a more free-flowing expression of ideas and emotions (97, 99, 100), and thus, greater openness. Further research into this complex phenomenon in persons with FTD may provide new insights into the neural basis of openness and ultimately inform new strategies for the treatment and care of these individuals.

## Conclusion

Openness is a complex construct that encompasses multiple dimensions, but examining the neurobiological basis of openness improves our understanding of the cognitive components of this personality trait. Structural, functional, and lesion studies converge to suggest that connectivity among specific brain networks supports trait openness; specifically, the interaction among (1) reward systems, mediated both by neurotransmitters like dopamine and serotonin and brain structures like the ventromedial prefrontal cortex (vmPFC); (2) frontal and parietal structures in the default mode network (DMN), supporting interpersonal perspective taking, self-reflection, and abstraction; and (3) dorsolateral prefrontal cortex (dlPFC) structures in the executive control network (ECN) that mediate cognitive flexibility and problem-solving. These three brain systems interact synergistically to support openness by increasing mental flexibility, reward responsiveness and novelty-seeking, and the ability to incorporate creativity into thought processes, decision-making, and behavior.

While creativity is typically associated with openness as a positive behavior, in some cases, it can also be associated with dysfunction or pathology. Reductions in openness are often seen in persons with brain disease and injury, particularly those affecting the frontal and temporal lobes. These changes are likely associated with alterations in neurotransmitter levels as well as brain structure and connectivity. The exact neurobiological mechanisms underlying unleashed creativity in FTD remain unclear, though disruptions in the frontotemporal networks critical for the integration of sensory, emotional, and cognitive information may lead to a breakdown in inhibitory processes that normally suppress creative expression, resulting in the emergence of novel and innovative ideas.

Clearly, further research is needed to understand the neuroanatomical basis of openness at a more granular level. With a richer and more precise understanding of the mechanisms underlying openness, better interventions could be developed to augment this highly positive trait, enhancing an individual’s receptiveness to new experiences, ideas, perspectives, and values, and thus promoting many aspects of their brain health. From a policy interventional perspective, these links between brain health and openness suggest that fostering openness as a personality trait has the potential for far-reaching benefits across the lifespan on both personal and societal levels. Promoting openness within educational systems and workplaces can shape environments that nurture curiosity, creativity, and a willingness to embrace new ideas. Successful interventions could contribute to the development of individuals who are adaptable, innovative, and open-minded, ultimately leading to better outcomes in education, workforce productivity, social cohesion, and personal brain health and mental well-being.

## Author contributions

The manuscript benefited from the collective input of all authors during the conceptualization stage having all authors taking part in developing the ideas for this manuscript. KR and MR played a significant role in designing and structuring the paper. The initial draft was written by MR, who served as the first author, while the other coauthors contributed by reviewing and making edits. KR supervised the work and contributed to the writing, reviewing, and editing processes. All authors contributed to the article and approved the submitted version.

## Conflict of interest

The authors declare that the research was conducted in the absence of any commercial or financial relationships that could be construed as a potential conflict of interest.

## Publisher’s note

All claims expressed in this article are solely those of the authors and do not necessarily represent those of their affiliated organizations, or those of the publisher, the editors and the reviewers. Any product that may be evaluated in this article, or claim that may be made by its manufacturer, is not guaranteed or endorsed by the publisher.
